# ERβ mediates sex-specific protection in the *App-NL-G-F* mouse model of Alzheimer’s disease

**DOI:** 10.1186/s13293-025-00711-w

**Published:** 2025-04-29

**Authors:** Aphrodite Demetriou, Birgitta Lindqvist, Heba G. Ali, Mohamed M. Shamekh, Mukesh Varshney, Jose Inzunza, Silvia Maioli, Per Nilsson, Ivan Nalvarte

**Affiliations:** 1 Department of Neurobiology, Care Sciences and Society, Division of Neurogeriatrics, Karolinska Institutet, Visionsgatan 4, J9:20, 171 64 Solna, Sweden; 2 Department of Biosciences and Nutrition, Karolinska Institutet, 141 57 Huddinge, Sweden; 3 Department of Laboratory Medicine, Karolinska Institutet, 141 52 Huddinge, Sweden; 4 Department of Biochemistry, Faculty of Veterinary Medicine, Assiut University, Assiut, 71526 Egypt

**Keywords:** Estrogen receptor beta, APP knock-in, Sex differences, Alzheimer’s disease, Microglia, Amyloidosis, Sex hormone

## Abstract

**Background:**

Menopausal loss of neuroprotective estrogen is thought to contribute to the sex differences in Alzheimer’s disease (AD). Activation of estrogen receptor beta (ERβ) can be clinically relevant since it avoids the adverse systemic effects of ERα activation. However, very few studies have explored ERβ-mediated neuroprotection in AD, and no information on its contribution to the sex differences in AD exists. In the present study, we specifically explored the role of ERβ in mediating sex-specific protection against AD pathology in the *App*^*NL−G−F*^ knock-in mouse model of amyloidosis, and if surgical menopause (ovariectomy) modulates pathology in this model.

**Methods:**

We treated male and female *App*^*NL−G−F*^ knock-in mice with the clinically relevant and selective ERβ agonist LY500307. A subset of the females was ovariectomized prior to treatment. Y-maze and contextual fear conditioning tests were used to assess memory performance, and biochemical assays such as qPCR, immunohistochemistry, Western blot, and multiplex immunoassays, were used to evaluate amyloid pathology.

**Results:**

We found that Female *App*^*NL−G−F*^ mice had higher soluble Aβ levels in cortex and hippocampus than males and more activated microglia. ERβ activation protected against amyloid pathology and cognitive decline in both male and female *App*^*NL−G−F*^ mice. Although ovariectomy increased soluble amyloid beta (Aβ) in cortex and insoluble Aβ in hippocampus, as well as sustained neuroinflammation after ERβ activation, it had otherwise limited effects on pathology. We further identified that ERβ did not alter APP processing, but rather exerted its protection at least partly via microglia activation in a sex-specific manner.

**Conclusion:**

Combined, we provide new understanding to the sex differences in AD by demonstrating that ERβ protects against AD pathology differently in males and females, warranting reassessment of ERβ in combating AD.

**Supplementary Information:**

The online version contains supplementary material available at 10.1186/s13293-025-00711-w.

## Highlights

- We used the clinically more relevant *App*^*NL−G−F*^ mouse model of amyloidosis to compare pathology between males and females, as well as the sex-specific neuroprotective effects of estrogen receptor beta (ERβ).
- We found that females had more activated microglia and that ERβ protects against cognitive impairment and amyloid pathology in both sexes.
- ERβ did not alter APP processing, but rather modulated microglia function in a brain-region and sex-specific manner, with the strongest effects in males.
- Ovariectomy increased amyloid beta levels and sustained neuroinflammation but had generally, in contrast to earlier studies in other AD models, limited overall effects on AD pathology.

**Supplementary Information:**

The online version contains supplementary material available at 10.1186/s13293-025-00711-w.

## Plain language summary

About two-thirds of all Alzheimer’s disease (AD) patients are women. Although the reason for this sex difference is likely multifaceted, sex hormones are believed to be involved. The female sex hormone estrogen is known to mediate neuroprotection and loss of estrogen during the menopausal transition is believed to be a risk factor for AD. However, there is a gap in knowledge on how estrogenic neuroprotection occurs and if this neuroprotection is similar in men and women. In the current study, we specifically focused on one estrogen receptor, ERβ, and its role in mediating protection in a clinically relevant mouse model of AD and asked if there are any differences in this protection between male and female AD mice. Such information is of importance if proposing clinical trials targeting ERβ, which unlike targeting the ubiquitous estrogen receptor alpha (ERα), is not associated with adverse systemic effects. We found that ERβ activation indeed protects against amyloid plaque buildup and cognitive impairment in both males and females. Interestingly, this neuroprotection appeared to work differently in different brain regions and affected neuroinflammation and microglia immune cell function differently in males and females. Surgical menopause (ovariectomy) increased amyloid levels, which was counteracted by ERβ activation, and sustained high neuroinflammation but had otherwise limited effect on pathology. We provide the first study comparing ERβ-mediated protection on AD pathology in males and females, highlighting important sex differences that should be considered when proposing ERβ as a target to combat AD.

**Supplementary Information:**

The online version contains supplementary material available at 10.1186/s13293-025-00711-w.

## Introduction

Over recent years, an increasing number of studies have suggested that the female sex hormone estrogen (E2) elicits neuroprotective functions, which are lost upon menopause, and that this loss may at least partly account for the increased female prevalence of Alzheimer’s disease (AD) [1–3]. Indeed, bilateral oophorectomy has been identified as a possible risk factor for dementia [4–6]. However, since all aged women enter menopause, but not all get dementia, other risk factors must exist that interact with lower circulating E2 levels.

Three types of estrogen receptors are found in the brain, estrogen receptor alpha (ERα), beta (ERβ), and the G-protein coupled estrogen receptor (GPER1). While ERα is highly expressed in hypothalamus to regulate functions related to reproduction, the roles of GPER1 and ERβ are less clear, and all three receptors are expressed in regions important for cognitive behavior such as the cortex and hippocampus [1]. Previous studies have proposed ERβ to be of particular interest as a possible therapeutic target in mediating neuroprotection since its activation is, unlike that of ERα, not associated with adverse health effects [7]. In the context of AD, ERβ has been suggested to play a multifaceted role in neuroprotection and neuronal survival [8–12]. However, variations between studies have led to inconclusiveness and there is a gap in knowledge of the exact contribution of ERβ to the sex-differences in AD. A limitation for increased understanding likely includes usage of different AD mouse models with no direct comparison between the sexes. In addition, different types of ERβ ligands with varying selectivity have been used with different results, adding to inconclusiveness.

In this study, we focus selectively on the role of ERβ in mitigating amyloid pathology in the *App*^*NL−G−F*^ knock-in mouse model that exhibits robust Aβ pathology (but without APP overexpression), neuroinflammation, synaptic alterations, and behavior impairment [13]. We evaluate the effect of the selective ERβ agonist LY500307 on AD pathology in male and female *App*^*NL−G−F*^ mice. LY500307 has a 12-fold higher selectivity for ERβ over ERα and 32-fold more functional potency, and since it binds ERα in opposite orientation thereby preventing ERα’s proliferative effects in reproductive organs, it is considered as probably the most clinically relevant ERβ agonist developed so far [14, 15]. In addition, LY500307 has passed first lines of toxicity and safety tests and is currently in phase 2 clinical trials for alleviation of perimenopausal depression (Clinical trials identifier: NCT03689543). Our data show that LY500307 protects against Aβ plaque buildup in cortex and hippocampus, as well as against cognitive deterioration in both male and female *App*^*NL−G−F*^ mice. Although ERβ activation does not affect APP processing, it modulates microglial and neuroinflammatory response in a largely sex- and brain-area-specific manner with stronger effects in males. Finally, we show that removal of systemic E2 by ovariectomy (surgical menopause) can increase amyloid levels and sustain neuroinflammation but with limited overall effects on AD pathology in *App*^*NL−G−F*^ females. Our data contribute to the increased understanding to the sex differences in AD and warrant further exploration of ERβ as a potential therapeutic target in AD.

## Material and methods

### Animals and treatments

Male and female *APP*^*NL−G−F*^ knock-in mice (carrying the Swedish [NL], Arctic [G] and Iberian [F] mutations in the humanized Aβ peptide [13]) were obtained from local breeding using the C57/BL6 J strain background. At 2.5 months of age, female mice were selected randomly for bilateral ovariectomy or sham surgery. Similarly, at 3 months of age male and female mice were randomly selected for LY500307 (0.35 mg/kg/day, Santa Cruz Biotechnology, Dallas, TX, USA), dissolved in vehicle solution (40% Captisol, [Cydex pharmaceuticals, Lawrence, KS, USA], 1% ethanol, and 59% 0.1 M PBS), or vehicle treatment (vehicle solution) through oral gavage administration. The treatment regimen was daily delivery over 7 days, followed by 7 days of rest. The resting period was included to avoid hormone-induced downregulation of ERβ gene expression [16]. This was repeated twice after which animals were subjected to behavior studies [2 days after last treatment and 2 days of rest between tests] and sacrificed at 5 months of age. For brain dissection, animals were deeply anesthetized with isoflurane followed by intracardial ice-cold 0.1 M PBS perfusion. Half brain was fixed in cold 4% paraformaldehyde and the hippocampus and cerebral cortex of the other half were snap-frozen for biochemical assays. All procedures were performed in accordance with approved ethical permits (ethical approval ID 407 and ID 2199–2021, Linköping’s animal ethical board).

### Behavioral tests

#### Contextual cued fear conditioning

A conditioning semi-transparent plexiglass chamber of 17 × 17x25 cm (l x w x h) with a stainless-steel grid floor (grid spaced 0.5 cm apart, Ugo Basile, Gemonio, Italy) surrounded by sound-attenuating grey chest was used for training and conditioning tests under a constant light (50 Lux) and background white noise (77 db). The chest was fitted with a light-sensitive camera over the chamber. The chamber was cleaned with 70% ethanol before each individual mouse test. The contextual fear conditioning test was performed over a span of 3 days, as previously described [17]. Briefly, on the conditioning day, mice were individually and randomly placed in the chamber and allowed to explore for 2 min before the onset of the conditional stimuli in the form of two sound exposures (65 db, 2000 Hz) 1 min apart lasting for 30 s each. During the last 2 s of each conditioning stimulus, the mice received a mild electric foot shock (0.5 mA). The conditioning ended 1 min after the last shock. The next day, the mice were subjected to the contextual test where they were placed back in the same chamber (context A) for 3 min but were not subjected to any sound stimuli or foot shock. On the third day, the mice were subjected to the cued test in which they were placed back in the chamber that had been fitted with different environment (checkered wall patterns and white bottom, context B). The mice were free to explore the chamber for 2 min (baseline) before the onset of the sound stimuli (cue tone, 65 db, 2000 Hz) for the rest 2 min without any foot shock (cue test). Mouse movement was traced by a computer-based video tracking system (ANY-Maze 6.3 software, Stoelting, Dublin, Ireland). The freezing response was defined as the percentage of time a mouse remained motionless (divided into 30 s intervals).

#### Y-maze

Hippocampal-dependent spatial working memory and reference memory were analyzed using the standard Y-maze test. The Y-maze consisted of 3 arms (35 × 7 × 15 cm, made of non-reflective gray plastic, Noldus Wageningen, Netherlands) at 120° angle to each other. A random mouse from each test group was placed in the center of the maze, and the 5 min trial started when the experimenter was out of the room of the maze to allow uninterrupted movement of the animal. Both manual and automated recording (using EthoVision XT, Noldus, Wageningen, Netherlands) of number of entries into each arm was used to calculate the percent spontaneous alterations. Alternations were considered completed when a mouse performed successive entries into three different arms. The threshold for number of arm visits for data to be included was set to 10, and threshold for considering an arm visit was set to when at least half the mouse (excluding the tail) crossed the arm entry border. Percentage alternations were calculated as [total alterations/(# arm entries – 2)]. The Y-maze was cleaned with 70% ethanol before each individual mouse test.

### Immunohistochemical and histochemical analyses

4 µm thick paraffin-embedded sagittal mouse brain sections were fixed on glass slides, hydrated, followed by heat-induced antigen retrieval in a pressure steamer at 121 °C for 20 min, followed by 15 min permeabilization with 0.5% Triton-X 100 (Millipore, Burlington, MA, USA) and blocking using 10% Horse Serum (ThermoFisher Scientific, Waltham, MA, USA), 0.1% Tween-20 (Millipore) in 0.1 M PBS for 1 h at 37 °C. Following blocking the slides were immunostained over-night at 4 °C with antibodies specific to Aβ (1:2000 dilution, 82E1, IBL-Tecan, Männedorf, Switzerland), Iba1 (1:300, ab178846; and 1:300 ab225260, both from Abcam, Cambridge, UK), GFAP (1:300, GA5 Alexa Fluor_488_-labeled, Millipore), CD68 (1:300, Ab283654, Abcam), and/or ERβ (1:5000, PP-PPZ0506-00, R&D Systems, Minneapolis, MN, USA) (Supplemental Table 1). For antibodies raised in mice, we used 1 × mouse-on-mouse IgG blocking solution (ThermoFisher Scientific) prior to antibody incubation. Secondary antibodies were Alexa Fluor_488_, Alexa Fluor_568_ (both from ThemoFisher Scientific). To reduce autofluorescence, the sections were incubated in 1 mM CuSO_4_ diluted in 50 mM ammonium acetate for 15 min. Nuclear staining was with 300 nM DAPI (ThermoFischer Scientific) for 10 min, prior to mounting. To visualize amyloid plaques, we used 1 × AmyloGlo stain (Biosensis, Thebarton, Australia) supplemented to the secondary antibody solution. ABC-HRP kit and Impact-DAB (both from Vector Laboratories, Newark, CA, USA) were used for immunohistochemical staining according to manufacturer’s recommendations. Immunofluorescence images were captured using an AxioPlan-2 fluorescent microscope (Carl Zeiss, Oberkochen, Germany) and the Zeiss AxioVision 4.0 software (Carl Zeiss). Image analysis was performed on at least 3 sections per mouse using the ImageJ software (NIH, Bethesda, MD, USA) and setting image threshold and counting was as described previously [18]. For each acquired image, the image lookup table (LUT) was kept linear and covered the whole image data. Association of microglia to plaques were quantified by counting number of microglia within 20 µm radius of plaque edge.

### Aβ and cytokine profile immunoassays

Frozen cortical and hippocampal tissues were thawed and homogenized in ice-cold TBS buffer (50 mM Tris–HCl, pH 7.6, 150 mM NaCl, and protease inhibitor cocktail (Roche, Basel, Switzerland)). The homogenates were centrifuged at 24 000 × g for 45 min at + 4 °C, yielding a soluble fraction (supernatant) and an insoluble fraction (pellet). The pellets were solubilized by resuspension in 6 M Guanidine-HCl and sonication using a water-bath sonicator (Bioruptor, 5 min max output (Diagenode, Denville, NJ, USA)). Soluble pellets were centrifuged at 24 000 × g for 45 min at + 4 °C and the supernatant (defined as insoluble fraction) was diluted in TBS to yield 0.5 M Guanidine-HCl. Similarly, Guanidine-HCl was added to the soluble fractions to yield a concentration of 0.5 M Guanidine-HCl. Total protein concentration was determined using the BCA Protein Assay (ThermoFisher Scientific) or the Coomassie Protein Assay reagent (Sigma-Aldrich, St Louis, MO, USA). Quantification of Aβ_1–40_ and Aβ_1–42_ in soluble and insoluble fractions was performed using the EZHS-SET ELISA kit, following manufacturer’s instructions (Millipore) and read on a Tecan plate spectrophotometer. Proinflammatory cytokine profiling on soluble fractions were performed using the V-PLEX proinflammatory panel 1 (mouse) kit (Mesoscale Discovery, Rockville MD, USA) on soluble brain fractions according to manufacturer’s instructions. The kit allows multiplex quantification of IFN-γ, IL-1β, IL-2, IL-4, IL-5, IL-6, IL-10, CXCL1 (KC/GRO, keratinocyte-derived chemokine/growth-related oncogene), IL12p70, and TNF-α. Samples were read on the MESO QuickPlex SQ120 reader and data were analyzed using the Discovery Workbench 4.0 software (both from Mesoscale Discovery). The concentration of each cytokine in the tissue lysates was normalized with the total protein concentration of the respective sample.

### Western blot

Cortical and hippocampal tissue were homogenized in ice-cold 4 × PIPES buffer pH 6.8 (40 mM Piperazine-1,4-bis(2-ethanesulfonic acid), 1.2 M Sucrose, 0.4 M NaCl, 27 mM MgCl_2_, and 1 × protease inhibitor cocktail, all from Sigma). Cell debris were pelleted and supernatants were centrifuged at 24 000 × g for 45 min at + 4 °C. The pellet was resuspended in a low volume of 4 × PIPIES buffer, and protein concentration was measured and adjusted to 2.5 mg/ml. 100 µg protein was incubated at 37 °C for 30 min followed by chloroform–methanol protein precipitation. In brief, 600 µl of cholorform:methanol in ratio 2:1 was added to the protein mixture and incubated for 30 min at room temperature (RT) under agitation followed by centrifugation and phase separation at 24,000×*g* for 15 min at RT. The intermediate was isolated, resuspended in 600 µl cholorform:methanol 1:2 and incubated for 60 min at RT under agitation. The protein was precipitated by centrifugation at 24,000×*g* for 15 min at RT, supernatant was removed, and pellet was let to dry. The protein pellet was resuspended in SDS-loading buffer to yield 3 mg/ml. In brief, 10–30 µg of protein were loaded on 4–20% gradient SDS-PAGE gels and proteins were transferred to a PVDF membrane. After blocking the membrane was subjected to antibody against Aβ1–16 (6E10, BioLegend), APP N-terminus (22 C11, Millipore), APP C-terminus (A8717, Sigma-Aldrich) and antibody against β-Actin (AC-15, Millipore). Detection was performed using ECL substrate (ThermoFisher) and exposure to light-sensitive films or CCD camera. Quantification of bands was performed using ImageJ software (NIH). All blots were processed in parallel.

### Real-time quantitative PCR analysis

Total RNA from cells or tissue was extracted using the RNeasy plus mini kit, RNeasy plus micro kit or Allprep DNA/RNA kit (Qiagen) according to manufacturer’s instructions, and RNA concentrations and quality were determined with NanoDrop (ThermoFisher Scientific). Complementary DNA was synthesized using SuperScript IV VILO Master Mix cDNA synthesis kit (ThermoFisher Scientific). The qPCR reaction contained 5 or 10 ng of cDNA, exon-exon spanning primers (500 nM), and KAPA SYBR Fast qPCR master mix (Sigma-Aldrich) or using TaqMan assays (Supplemental Table 2) and TaqMan Fast Advanced Master Mix (Applied Biosystems) and was performed on an ABI 7500 fast thermal cycler (Applied Biosystems) according to manufacturer's instructions. Expression relative to housekeeping gene was calculated using the ΔCt method.

### Statistical analysis

Results are expressed as means ± SD. The statistical analyses were performed using GraphPad Prism 9.02 software (GraphPad Software, San Diego, USA). Data were tested for equal variance by *F*-tests. Unpaired two-tailed Student’s t-tests were used to compare between two groups. Unless stated otherwise, multiple group analyses were performed by two-way or three-way analysis of variance (ANOVA), followed by uncorrected Fisher’s LSD test or corrected post-hoc tests for multiple comparisons as indicated in figure legends. Significance level was set at < 0.05 (*P < 0.05, **P < 0.01, ***P < 0.001, ****P < 0.0001). All analyses are based on at least 3 biological replicates.

## Results

### ERβ expression in the mouse cortex and hippocampus

Since estrogen (E2) has been ascribed neuroprotective properties [1–3], we sought to explore if selective activation of the estrogen receptor beta (ERβ, *Esr2* gene product), a more clinically relevant target than ERα, can be protective against amyloid-related pathology in the *App*^*NL−G−F*^ mouse model of AD. Since the expression of ERβ in the brain has been questioned due to poor antibody specificities to ERβ, we first performed immunohistochemical analysis using a validated ERβ antibody in wild-type (WT) and ERβ knockout (*Esr2*-KO) mouse brains. This revealed scattered expression with both cytoplasmic and nuclear localization in several brain regions affected in AD, including the frontotemporal, primary motor, somatosensory, and visual cortices, as well as in the granule layers of CA2 and dentate gyrus (DG) of the hippocampus (Supplemental Fig. 1 A, B). Highest number of ERβ positive cells were seen in frontal and primary motor cortex, as well as in the hippocampus (Supplemental Fig. 1B). ERα (*Esr1*) and ERβ (*Esr2*) had similar expression between male and female mice in cortex and hippocampus although ERα expression was about 5–tenfold higher than ERβ in both brain regions, and ERα expression did not change upon ERβ loss (Supplemental Fig. 1C–F).

**Fig. 1:**
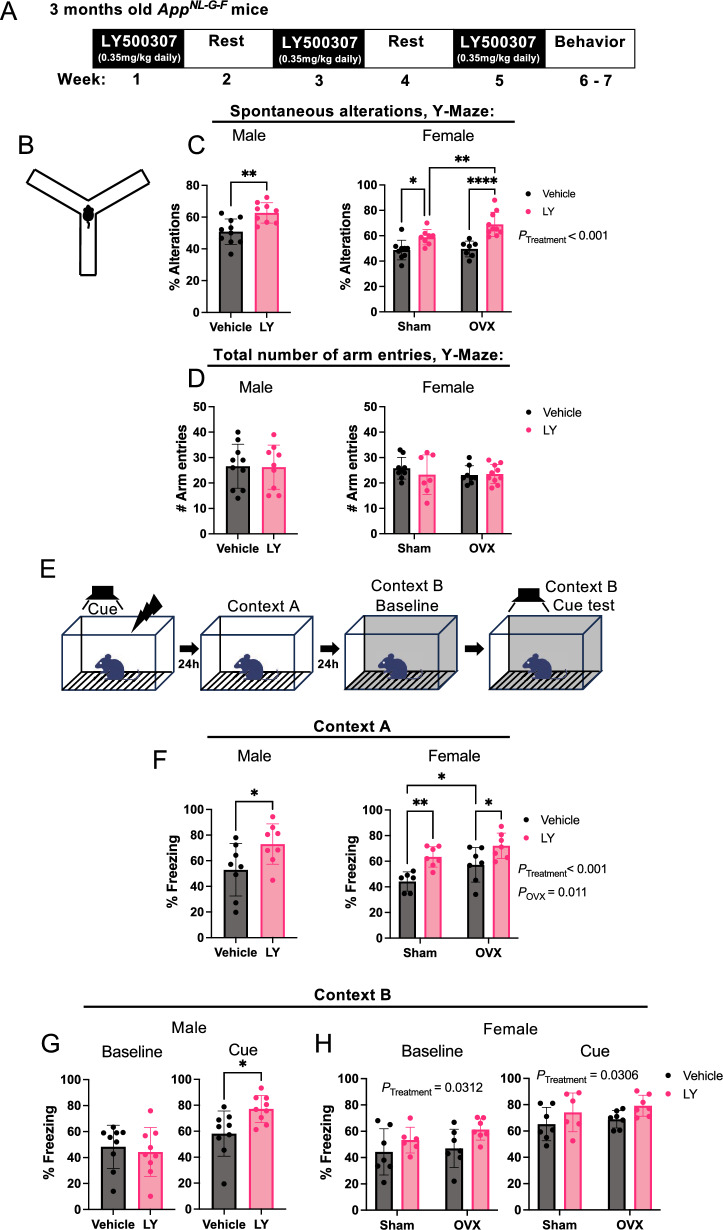
ERβ activation improves cognitive behavior in *App*^*NL−G−F*^ male and female mice. **A** Treatment regime of *App*^*NL−G−F*^ mice. **B** Representative image of Y-maze arena. **C** Percent Y-maze arm alterations and **D** total number of arm entries of male (left) and female (right) *App*^*NL−G−F*^ mice treated with vehicle or ERβ agonist LY500307 (LY) (n = 7–10). **E** Diagram showing the fear conditioning paradigm. **F** Percent context-associated freezing time of male (left) and female (right) *App*^*NL−G−F*^ mice (n = 6–9) in the contextual fear conditioning test. Cued-associated freezing time in the contextual fear conditioning test of **G** male and **H** female *App*^*NL−G−F*^ mice before cue (baseline) and upon cue (tone) in a different cage context (n = 6–9). Female mice were either ovariectomized (OVX) or sham operated (Sham). * *P* < 0.05, ** *P* < 0.01, *** *P* < 0.001. Unpaired t-test was used for males and 2-way ANOVA for females followed by uncorrected Fisher’s LSD test for multiple comparisons. Overall significant main effects of treatment or OVX are indicated

### ERβ activation protects against cognitive deficits and Aβ_42_ deposition in ***App***^***NL−G−F***^ mice

To study the effect of ERβ activation on AD pathology we treated *App*^*NL−G−F*^ male and female mice with the selective ERβ agonist LY500307 daily through oral gavage (0.35 mg/kg) every other week over 5 weeks, starting at 3 months of age. A subset of female mice was ovariectomized (OVX) 2 weeks prior to treatment to study the influence of loss of circulating E2 (Fig. 1A). At the end of the treatment, the mice were subjected to memory tests, where the mice treated with LY500307 (LY) performed better than vehicle-treated mice in the Y-maze spatial memory test (Fig. 1B, C). Interestingly, we did not observe any adverse effect of OVX on spatial memory, rather a better performance in combination with LY (Fig. 1C). There were no effects on total arm entries (Fig. 1D). Associative memory was tested using the cued fear conditioning (FC) paradigm (Fig. 1E). Mice given LY performed better than vehicle-treated mice in the FC test, with longer episodes of freezing both during the contextual (Fig. 1F) and cued tests (Fig. 1G, H). Interestingly, LY-treated females also displayed increased freezing at baseline, even in the new context, suggestive of an overall better memory performance after LY treatment (Fig. 1H). Again, OVX did not have a negative effect on memory performance, oppositely, it slightly improved memory in the contextual FC test, although less significant compared to LY treatment (Fig. 1F). Overall, these results suggest that ERβ activation may indeed act neuroprotective in the *App*^*NL−G−F*^ model.

**Fig. 2:**
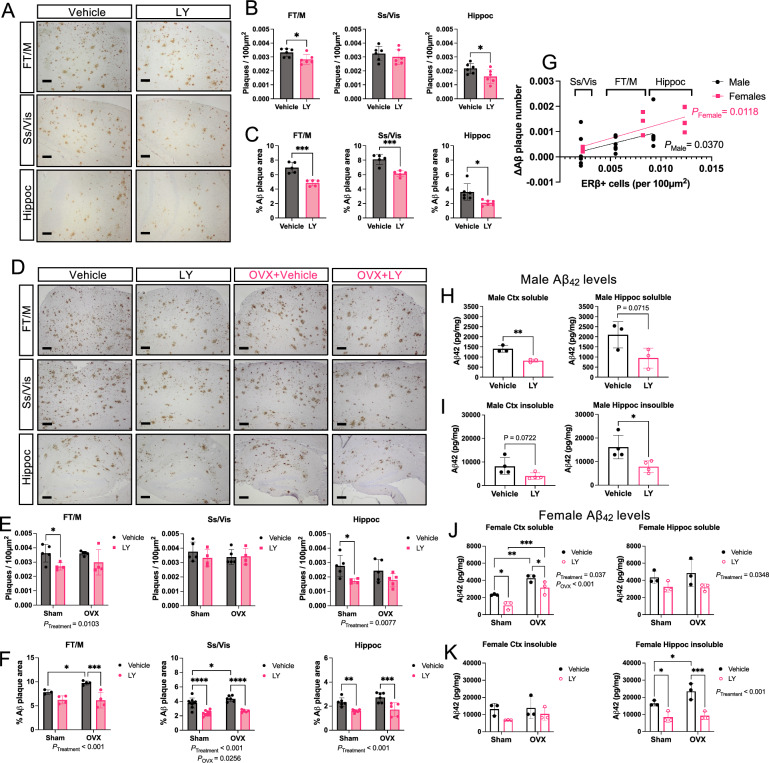
Less Aβ pathology in *App*^*NL−G−F*^ male and female mice after ERβ activation. **A** Immunohistochemical representation of amyloid plaques in frontal and motor cortex (FT/M), somatosensory and visual cortex (Ss/Vis) and hippocampus (Hippoc) of male *App*^*NL−G−F*^ mice after vehicle or LY treatment. **B** Quantification of number of plaques per 100 µm^2^ (n = 4–6) and **C** percent plaque area (n = 4–6) in male *App*^*NL−G−F*^ mice. **D** Similar as in **A**, immunohistochemical representation of amyloid plaques in different brain regions of female *App*^*NL−G−F*^ mice after vehicle or LY treatment. **E** Quantification of number of plaques per 100 µm^2^ (n = 4–5) and **F** percent plaque area (n = 4–9) in female *App*^*NL−G−F*^ mice. **G** Linear regression analysis comparing effect size from LY treatment (vehicle vs. LY) on number of Aβ plaques in relation to average number of ERβ positive cells per 100 µm^2^ in different brain regions of male and female mice (n = 4–6). **H** Soluble and (I) insoluble Aβ42 levels in male cortex (Ctx, left) and hippocampus (Hippoc, right) (n = 3–4). (J) Soluble and (K) insoluble Aβ_42_ levels in female cortex (left) and hippocampus (right) (n = 3). * *P* < 0.05, ** *P* < 0.01, *** *P* < 0.001, **** *P* < 0.0001. Unpaired t-test was used for males and 2-way ANOVA for females followed by uncorrected Fisher’s LSD test for multiple comparisons. Overall significant main effects of treatment or OVX are indicated. Scale bars = 100 µm

Next, we analyzed the effect of LY on amyloid pathology. LY-treated mice had generally lower number and smaller size of amyloid plaques in different cortical regions and in hippocampus in both male (Fig. 2A–C) and female (Fig. 2D–F) *App*^*NL−G−F*^ mice. When taking number of ERβ positive cells into account in those regions, we could observe that the largest effect size of LY treatment in lowering Aβ plaques overlapped with highest levels of ERβ positive cells in those regions (Fig. 2G, Supplemental Fig. 1B). OVX did not have any effect on number of plaques, but slightly (but significantly) increased existing plaque area in visual and somatosensory (Vis/Ss) cortex (Fig. 2E, F). In line with these results, the levels of soluble and insoluble neurotoxic amyloid beta (Aβ_42_) were overall lower in cortex and hippocampus in both male (Fig. 2H, I) and female (Fig. 2J, K) *App*^*NL−G−F*^ mice after LY treatment, although it did not reach statistical significance for hippocampal soluble and cortical insoluble Aβ_42_ in male mice and no statistical significance for cortical insoluble Aβ_42_ levels in females. Interestingly, OVX increased Aβ_42_ levels in the cortex, while having no effects on Aβ_42_ levels in other brain areas. Aβ_40_ levels were similar to Aβ_42_ levels (Supplemental Fig. 2 A–D) and Aβ_42_/Aβ_40_ ratio did not differ with LY treatment, although there was an overall increase in soluble Aβ_42_/Aβ_40_ ratio in female cortex upon OVX (Supplemental Fig. 2F). Furthermore, female mice had generally higher levels of soluble Aβ_42_ levels compared to male mice (Supplemental Fig. 2G, H). These data suggest that ERβ activation reduces Aβ levels and plaque load in both male and female *App*^*NL−G−F*^ mice, but sex differences exist, and that OVX can worsen amyloid pathology although differently in in different brain regions.

### Effect of ERβ on APP processing

**Fig. 3:**
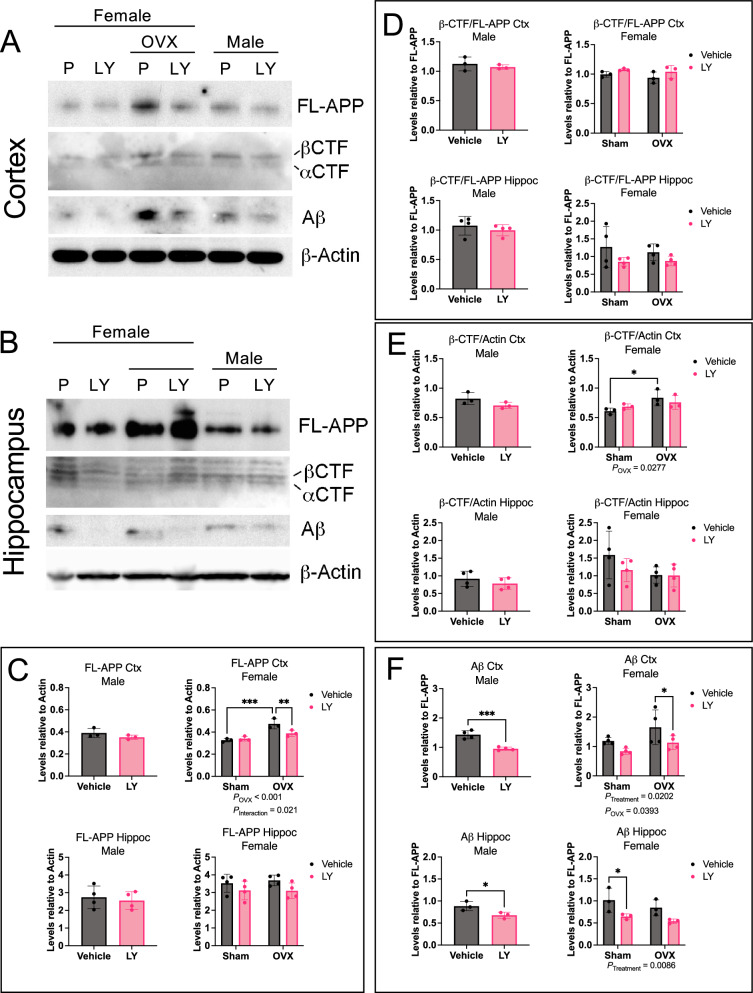
ERβ activation does not alter APP processing. Western blot analysis of full-length APP (FL-APP), β-CTF and α-CTF, Aβ peptide, and β-actin in **A** cortex and **B** hippocampus of female and male *App*^*NL−G−F*^ mice after vehicle (V) or LY treatment, as well as after sham surgery or ovariectomy (OVX in females). Quantification of **C** FL-APP relative to β-actin, **D** β-CTF relative to FL-APP, **E** β-CTF relative to actin, and **F** Aβ relative to FL-APP in male (left), and female (right), cortex (Ctx) (top), and hippocampus (bottom) (n = 3–4). * *P* < 0.05, ** *P* < 0.01, *** *P* < 0.001. Statistical significance was determined using unpaired t-test for males and 2-way ANOVA for females followed by uncorrected Fisher’s LSD test for multiple comparisons. Overall significant main effects of treatment or OVX are indicated

To explore if reduced Aβ_42_ levels in LY-treated mice was a consequence of lower APP levels or a shift from amyloidogenic β-secretase processing to non-amyloidogenic α-secretase processing, we analyzed the levels of full-length APP (FL-APP) and processed APP fragments. Western blot analysis revealed that LY-treatment of male mice had no effect on FL-APP levels in cortex nor in hippocampus (Fig. 3A–D). However, FL-APP was significantly increased in female cortex upon OVX and decreased upon LY-treatment of OVX females (Fig. 3A–C). LY treatment did not result in any difference in C-terminal fragment β-CTF levels relative to FL-APP (Fig. 3D), but β-CTF was increased upon OVX relative to β-actin levels (Fig. 3E), suggesting α- or β-secretase activities are not altered by ERβ activation (Fig. 3A, B, D). In addition, the expression of *App* or processing enzymes (*Bace1, Psen1,* and *Adam10)* were not altered by LY treatment or OVX (Supplemental Fig. 3C–J), further suggesting that ERβ does not modulate APP processing, although OVX increased APP protein levels in female cortex (Fig, 3 A, C). Finally, we again observed lower total Aβ levels upon LY treatment in both male and female cortex and hippocampus and an interesting increase in cortex upon OVX (Fig. 3A, B, F), similar to what is seen in Fig. 2J. These data suggest that ERβ does not directly modulate APP processing but may rather be involved in the clearance of Aβ.

### Effect of ERβ on glial cells in ***App***^***NL−G−F***^ mice

**Fig. 4:**
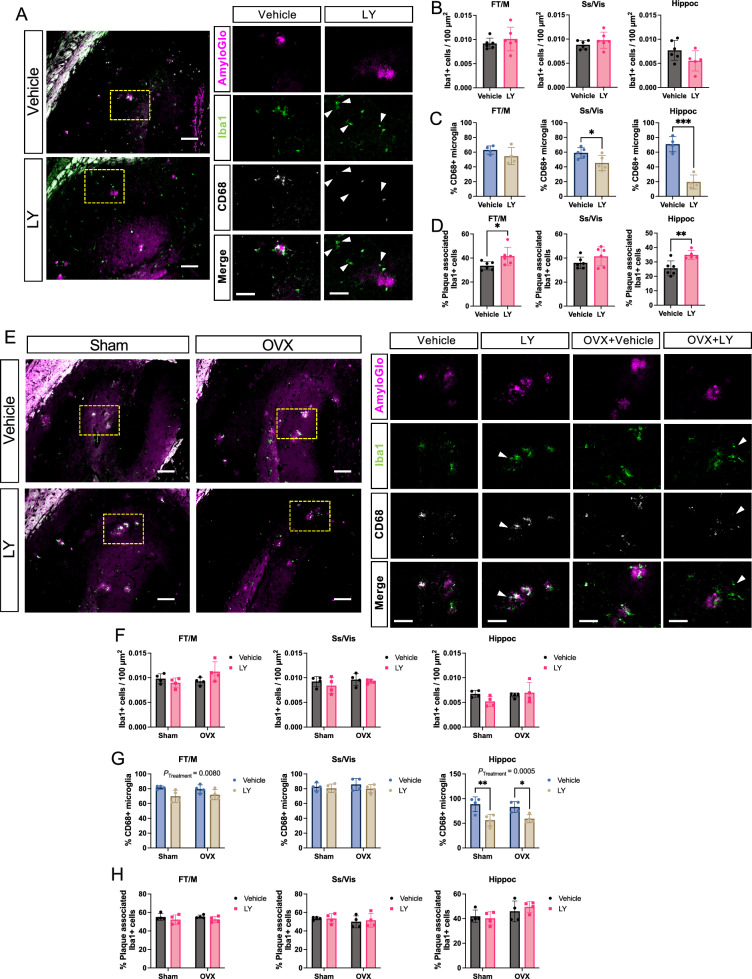
ERβ activation modulates microglia activation in a sex-specific manner in *App*^*NL−G−F*^ mice.** A** Representative immunofluorescence images of male *App*^*NL−G−F*^ hippocampus stained with the amyloid stain AmyloGlo (magenta), Iba1 (green), and CD68 (white) after vehicle or LY treatment. Yellow dotted area (left) indicates magnified region of interest (right). Arrowheads indicate microglia with lower CD68 levels. Scale bar 100 µm (left) and 50 µm (right). Quantification in male *App*^*NL−G−F*^ mice of **B** number of Iba1 cells per 100 µm^2^ (n = 5–6), **C** percent CD68 +, Iba1 + double positive cells (n = 4–5), and **D** percent microglia within 20 µm radius of plaque edge (n = 5–6). **E** Representative immunofluorescence images of female *App*^*NL−G−F*^ hippocampus stained with AmyloGlo (magenta), Iba1 (green), and CD68 (white) after vehicle or LY treatment, as well as after sham surgery or ovariectomy (OVX). Yellow dotted area (left) indicates magnified region of interest (right). Arrowheads indicate microglia with lower CD68 levels. Scale bar 100 µm (left) and 50 µm (right). Quantification in female *App*^*NL−G−F*^ mice **F** number of Iba1 cells per 100 µm^2^ (n = 4), (**G** percent CD68 +, Iba1 + double positive cells (n = 4), and **H** percent plaque-associated microglia (n = 4). * *P* < 0.05, *** *P* < 0.001. Unpaired t-test was used for males and 2-way ANOVA for females followed by uncorrected Fisher’s LSD test for multiple comparisons. Overall significant main effects of treatment are indicated

Astrocytes and microglia take active part in amyloid pathogenesis, including Aβ clearance [19]. Therefore, we sought to investigate the impact of ERβ activation on astrocytic and microglial response. We could not detect any major effects on astrogliosis in male and female mice treated with LY or in OVX females (Supplemental Fig. 4 A–D). Similarly, we did not see any difference in microglia numbers upon LY treatment in male *App*^*NL−G−F*^ mice (Fig. 4A, B, Supplemental Fig. 5A). However, LY treatment significantly reduced number of activated CD68 + microglia, especially in the male hippocampus (Fig. 4A, C), and we also observed that LY treatment promoted microglia association to amyloid plaques in male frontal/motor cortex and in hippocampus (Fig. 4D). In female *App*^*NL−G−F*^ mice, LY treatment had less effect on microglia (Fig. 4E–H, Supplemental Fig. 5B) compared to male mice, and we could detect a slight, but significant, effect of LY on lowering the number of activated CD68 + microglia in frontal/motor cortex and in hippocampus (Fig. 4G), but no effect on microglia association to plaques (Fig. 4H). We could also not detect any significant effect of OVX on microglia numbers or activation. However, female mice had an overall increased number of CD68 + microglia compared to male mice, and overall increased number of plaque-associated microglia, but less response to LY (Supplemental Fig. 5C, D).

**Fig. 5:**
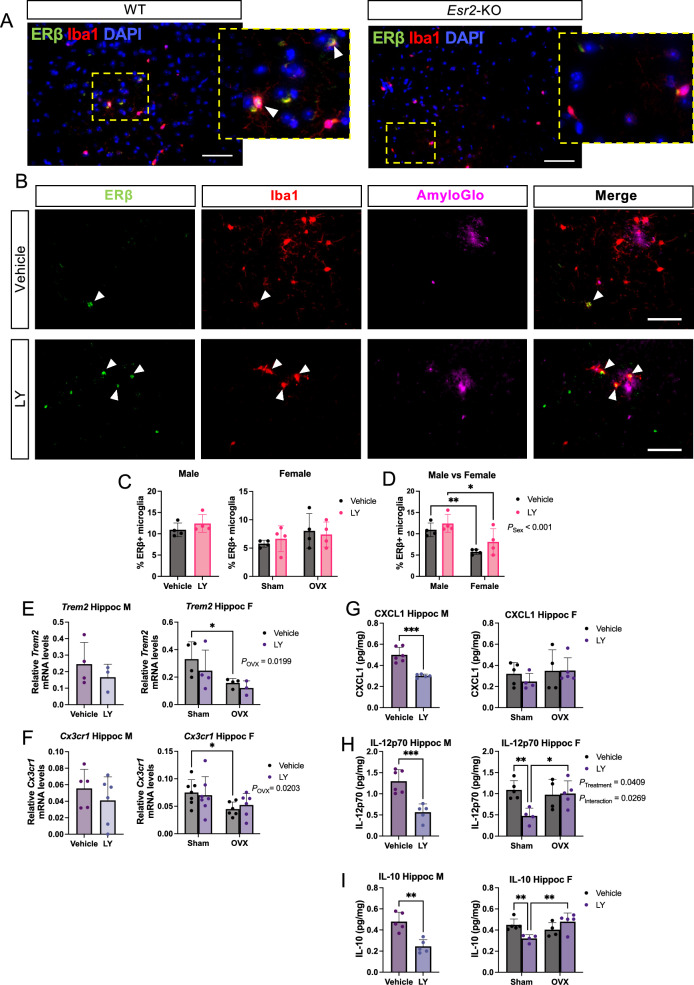
Microglial and proinflammatory markers are altered upon ERβ activation in a sex-specific manner.** A** Representative immunofluorescence image of ERβ and Iba1 co-staining (arrowheads) in WT (left) and *Esr2*-KO (right) male cortex (dotted rectangle: magnified area), and **B** in male *App*^*NL−G−F*^ cortex upon vehicle or LY treatment (scale bars = 50 µm). **C** Quantification and **D** comparison of percent ERβ positive microglia in male and female *App*^*NL−G−F*^ brains (cortex and hippocampus) upon OVX and/or LY treatment (n = 4). Expression of the proresolving microglial markers **E** *Trem2*, and **F** *Cx3cr1* relative to housekeeping gene *Rplp0* in male (left) and female (right) *App*^*NL−G−F*^ hippocampus after vehicle or LY treatment, as well as after sham surgery or OVX in females (n = 3–7). Multiplex ELISA analysis of the inflammatory markers **G** CXCL1 (KC/GRO), **H** IL-12p70, and **I** IL-10, in male (left) and female (right) *APP*^*NL−G−F*^ hippocampus after vehicle or LY treatment, as well as after sham surgery or ovariectomy (OVX in females) (n = 4–6). * *P* < 0.05, ** *P* < 0.01, *** *P* < 0.001. Unpaired t-test was used for males and 2-way ANOVA for females followed by uncorrected Fisher’s LSD test for multiple comparisons. Overall significant main effects of treatment or OVX are indicated

We next analyzed the expression of ERβ in microglia. To avoid false positives, we used ERβ knockout (*Esr2*-KO) mice as staining control (Fig. 5A). Although some microglia showed ERβ positive staining, most microglia were ERβ negative (Fig. 5A). In *App*^*NL−G−F*^ mice, plaque-associated microglia were both ERβ positive and negative and LY treatment had no overall effect on ERβ-Iba1 co-expression in any brain region examined (Fig. 5B, C). However, there were slightly but significantly more ERβ + microglia in male compared to female *App*^*NL−G−F*^ brains (Fig. 5D), which could possibly explain the higher responsiveness of male microglia to LY-treatment (in Fig. 4).

Studying the expression of two specific microglial markers associated with anti-inflammatory and pro-resolving responses, *Trem2* and *Cx3cr1*, we could not detect any effect of LY in either male or female hippocampus (Fig. 5E–H). However, OVX lowered expression of these markers. No difference in these markers was observed in cortex (data not shown). Using a detection-panel of proinflammatory cytokines we observed significantly lower levels of CXCL1 (KC/GRO) in the hippocampus of LY-treated male mice (Fig. 5H) and of IL-12p70 in both male and female LY-treated mice (Fig. 5H). Additionally, the level of the danger signal IL-10 was lower both male and female LY-treated mice (Fig. 5I), which may suggest less hyperinflammation [20, 21] upon LY treatment. Of these markers, IL-12p70 and IL-10 levels were sustained in LY-treated OVX females compared to vehicle-treated mice (Fig. 5H, I). Thus, these data suggest less neuroinflammation upon ERβ activation in both male and female *App*^*NL−G−F*^ mice and that OVX can bypass this effect of ERβ. In addition, it suggests that ERβ exhibit its neuroprotective effects likely through additional cell types than microglia, especially in females.

## Discussion

The neuroprotective properties of estrogen have been suggested to decrease after menopause [1–3]. ERβ can be a clinically relevant target to combat neurodegeneration and, unlike ERα, does not have any adverse systemic effects upon activation. In this study, we specifically explored the role of ERβ in mediating neuroprotection in the *App*^*NL−G−F*^ mouse model of AD, a model that unlike previous AD models circumvents the artefacts from APP overexpression, making it one of the more relevant models of human AD [13]. We show that selective ERβ activation with the clinically relevant LY500307 (LY) protects against amyloid pathology and memory deficits in *App*^*NL−G−F*^ mice. We also show that this neuroprotection is different between males and females, likely involving different cell types, and that ovariectomy (OVX) increases Aβ_42_ levels and sustains neuroinflammation but has otherwise a limited effect on overall pathology in *App*^*NL−G−F*^ mice.

Despite previous problems with ERβ antibody specificities, it is now clear that both ERα and ERβ are expressed in the adult cortex and hippocampus but in a scattered manner and at relatively low levels [1]. E2 has been ascribed general neuroprotective effects by protecting against apoptosis [22], sustaining mitochondrial health, and thereby regulating oxidative stress [2, 23, 24]. E2 also promotes neurogenesis and synaptic plasticity upstream of BDNF [25, 26] and WNT signaling [27, 28]. Although these pathways likely also contribute to estrogenic neuroprotection in AD, very few studies exist on the role of ERs in animal AD models, and none address possible sex differences in estrogenic neuroprotection. However, it has been shown that ERα activation protects against memory deficits in female APP/PSEN1 transgenic mice [29], and reduces Aβ accumulation in female 3xTg-AD transgenic mice [10]. Similarly, ERβ activation using dietary phytoestrogens (with various specificity to ERβ) lowers Aβ deposition and ameliorates cognitive deficits in female APP/PSEN1 mice [9, 30, 31] and in female [11] and male [32] 3xTg mice, which in part could be attributed to modulated BDNF and WNT signaling, and enhanced microglial phagocytosis [11, 31]. Importantly, a direct comparison between ERβ activation in male and female AD models has until now been missing, which has limited the neuroendocrinological understanding behind the sex-differences in AD.

Early loss of circulating estrogen and progesterone such as in early menopause or bilateral oophorectomy may be a risk factor for AD [4–6], and E2 supplementation could protect against this risk. Human data on such protective associations are limited and controversial [33]. However, animal studies using 3xTg-AD mice demonstrate that gonadectomy leads to increased Aβ accumulation and cognitive impairment, while estrogenic supplementation protects against these deficits [10, 32, 34–36]. Interestingly, similar protection was not seen in a study involving ovariectomized APP/PSEN1 mice [37], suggesting that inherent model characteristics may modulate estrogenic neuroprotection.

In the present study, selective activation of ERβ in the *App*^*NL−G−F*^ model, not only confirms previous studies in older AD transgenic models on ERβ’s protective effects [9, 11, 30, 31], but also identifies important new sex differences in ERβ mediated protection. However, in contrast to 3xTG AD models, OVX did not yield major effects on AD pathology in our study, in fact ovariectomized mice performed better in the contextual fear conditioning test (Fig. 1), which could possibly be related to secondary neurocognitive characteristics from OVX, such as anxiety or motility effects of OVX. However, there was no effect of OVX on total number of arm entries in the Y-maze test, which argues against decreased exploratory behavior and anxiety in OVX mice (Fig. 1D). Interestingly, some of the few clear effects of OVX were increased soluble and insoluble Aβ_42_ levels in cortex and hippocampus, respectively (Fig. 2J, K), which may be related to higher FL-APP levels in OVX mice (Fig. 3A, E) (with an interesting interaction between OVX and LY on cortical FL-APP levels (Fig. 3C)). Nevertheless, APP processing seemed to not be affected. OVX also decreased microglial *Trem2* and *Cx3 Cr1* expression in hippocampus (Fig. 5F, H), and sustained high IL-12p70 and IL-10 levels in LY-treated females, which may imply that basic sex hormone levels are needed for proper microglial function and ameliorates neuroinflammation in hippocampus of *App*^*NL−G−F*^ mice.

Overall, females had more hyperactivated microglia in all brain regions studied (Supplemental Fig. 5). ERβ activation markedly reduced microglia activation in both male and female mice, with the strongest effect in the male hippocampus, which was concomitant with decreased levels of proinflammatory markers (Fig. 5). This may mean that ERβ activation leads to less amyloidosis and therefore less neuroinflammation. However, ERβ activation also increased the number of plaque-associated microglia at least in male hippocampus (Fig. 4) (which also had more ERβ + microglia), which argues for a more direct and sex-dimorphic effect of ERβ on microglia.

More studies are needed to explain the impact of OVX in different brain cells and brain regions and its interaction with specific estrogen receptors. This is complex since OVX may have different functions in different brain regions, illustrated by how OVX modulates glucose metabolism differently in different brain regions [38]. In addition, E2 can be de-novo synthesized in different brain regions (including hippocampus and cortex) and in different cell types [39, 40], and our results on the OVX condition must be interpreted in the context of local de-novo synthesized E2. Similarly, it is likely that ERβ mediates brain region-specific functions through interactions with different cell type-specific factors. For example, ERβ (but not ERα) can regulate BDNF signaling in the female rodent brain in a region-specific manner [26]. Although LY treatments ameliorated OVX effects on amyloidosis, we must keep in mind that OVX affects all ER signaling, and does not necessarily affect local brain E2 production, so direct relationships between LY treatments and OVX cannot always be expected.

Our study also suggests that ERβ works differently with different effect sizes in cortex and hippocampus. Although ERβ mRNA expression levels were similar between cortex and hippocampus, a more detailed brain region analysis showed that the number of ERβ + cells was highest in frontal and primary motor cortex as well as in hippocampus (Supplemental Fig. 1 A, B), which overlapped with the brain regions with largest effects of LY on Aβ plaque numbers (Fig. 2G). Furthermore, as mentioned above, male microglia were more ERβ positive, which is in line with our observation that LY treatment has a larger effect on male microglia (Figs. 4, 5). A limitation of this study is that estrous cycle in *App*^*NL−G−F*^ mice was not compared to WT littermates, which means that the hormonal profile of *App*^*NL−G−F*^ mice is not known. However, since there are no reports on reproductive deficits in *App*^*NL−F*^ mice, we assume that these mice cycle normally with a comparable hormonal profile as WT mice. In addition, we have not observed any differences in litter sizes or number of litters in the *App*^*NL−G−F*^ colony compared to WT mice. Nevertheless, reproductive cycling could possibly be influenced by factors not reflected in litter size or numbers. This should be considered when interpreting the results of this study. Another limitation of this study is that we induce surgical menopause at a young reproductive age in a mouse model of aggressive amyloidosis, which may obscure effects of more natural chronological and endocrinological aging. Future studies of ERβ signaling in slow-progressing AD models (e.g., *App*^*NL−F*^ mice) are therefore needed. Another limitation of this study is the low number of biological replicates for some readouts. It is therefore important to assess our combined data to draw valid interpretations. Thus, combined, our study emphasizes the sex differences in ERβ's neuroprotection; in male mice this neuroprotection can be to a larger extent mediated through microglia, while in females other non-inflammatory processes downstream of ERβ activation appear to play a larger role. Autophagy may be such a process as suggested by Wei and coworkers [12].

## Conclusions

In conclusion, our study provides the first direct comparison of ERβ's sex-specific neuroprotective effects in an AD model. We show that this neuroprotection is not directly associated with altered APP processing, but rather to microglia function in a sex-specific manner, and that ovariectomy can increase Aβ levels and sustain neuroinflammation but with otherwise limited overall effects on AD pathology. Our research adds to the molecular understanding of the sex-differences in AD and warrants further studies on brain cell-specific effects of ERβ in male and female AD models and human AD patients.

## Supplementary Information

Additional file 1.

## Data Availability

No datasets were generated or analysed during the current study.
